# Nutritional impact of no-added sugar fruit puree consumption at different eating occasions: a modelling study on French children

**DOI:** 10.1017/S1368980024000739

**Published:** 2024-03-26

**Authors:** Romane Poinsot, Céline Richonnet, Florent Vieux

**Affiliations:** 1 MS-Nutrition, Faculté de Medecine la Timone, 27 boulevard Jean Moulin, Marseille, France; 2 Materne, 1 rue de la pépinière, Paris, France

**Keywords:** Fruit puree, Children, INCA3, Substitution, Fibres, Free sugars

## Abstract

**Objective:**

The recommended level of five fruits and vegetables per day is reached by a minority of French children. No-added sugars fruit puree (NASFP) can be consumed as a complement of fresh fruit to meet the recommendation for fruits and vegetables. The objective was to simulate the nutritional impact of an increase in consumption of NASFP among French children, together with a reduction in sweetened foods.

**Design:**

The study was conducted on French children aged 1–17 years. The simulation consisted in introducing NASFP on four different eating occasions (breakfast, lunch, snack and dinner) to reach one serving and removing the same serving of sweetened foods. Intakes in nutrients to favour, nutrients to limit and prevalence of adequacy to nutritional requirements were compared between observed and simulated diets in the whole sample and in five different age groups.

**Setting:**

France.

**Participants:**

Children from 1 to 17 years of age in the last available French representative dietary survey (INCA3).

**Results:**

Simulated diets were more nutrient-dense thanks to increases in nutrients to favour from NASFP (especially fibres, iodine, Se, and vitamin A and C) associated with reductions in energy and nutrients to limit (especially free sugars) coming from sweetened foods. Prevalence of adequacy increased from 2 to 14·5 points for fibres and from 4·5 to 12 points for free sugars according to age group and eating occasion.

**Conclusion:**

Promoting NASFP in replacement of sweetened products is a promising strategy to improve the nutritional quality of French children’s diet through a better adherence to national guidelines.

Inadequate fruit and vegetable consumption lead to an estimated 3·9 million deaths worldwide each year^(1)^. More precisely, 2 million deaths and 65 million disability-adjusted life years are attributable to low intake of fruits^(2)^ and suboptimal intake of fruits (i.e. less than 250 g) contributes to cardiovascular mortality^(3)^. Joint FAO/WHO Expert Consultation on Diet, Nutrition and the Prevention of Chronic Diseases recommends the intake of a minimum of 400 g of fruit and vegetables per d (excluding starchy tubers such as potatoes) for the prevention of chronic diseases, including heart disease, cancer, type 2 diabetes and obesity^(4)^, transcribes in five servings of at least 80 g a day in the French Public Health Policy for adults, including fresh, canned or frozen forms of fruits and vegetables^(5)^. For children, it is recommended to adapt the serving sizes to their age^(6)^. The ratio of two servings of fruit and three servings of vegetables may confer the highest longevity benefit and the optimal balance to reduce the risk of many chronic health conditions^(7)^, but this ratio is not subject to any official recommendation.

Yet in France, according to the 2014–2016 Health Study on Environment, Biomonitoring, Physical Activity and Nutrition (ESTEBAN), half of the children consumed less than 3·5 servings (i.e. less than 280 g) of fruits and vegetables per d^(8)^. The most recent national survey on the food consumption and eating habits of the French population (INCA3) conducted between 2014 and 2015 by The French Agency for Food, Environmental and Occupational Health Safety (Anses) showed that fruits and vegetables, along with cereals, are the main carriers of fibres in children^(9)^. Fruits and vegetables contributed to one-third of fibres intakes of youngest (0–10 years old) French children and more than one-quarter for teenagers (11–17 years old), including 9·2 % (7·3 % for teenagers) for fresh fruits and 6·2 % (1·7 % for teenagers) for fruits puree and fruits syrups. The insufficiency in fruits and vegetables consumption among children may thus contribute to inadequate intakes of fibres^(8)^. To the best of our knowledges, prevalence of adequacy to fibres recommendations among French children in INCA3 has not, to date, been published. According to ESTEBAN study, more than half of children between 6 and 10 years old consumed less than 15 g of fibres per d^(8)^. Given that the recommended fibre intake is at least 16 g between 7 and 10 years old^(10,11)^, it is likely that the prevalence of fibre adequacy in French children is low.

On the other hand, a systematic review has shown that out of twenty-four studied countries, France had the highest intake of SFA^(12)^. SFA represent between 14 and 15 % of the total energy intakes (EI) of children’s diet, although it should not exceed 12 %^(9)^. Concerning carbohydrates repartition, two-thirds of the children had intakes of simple sugars from sweetened products greater than 12·5 % of the total EI^(8)^. To reduce total sugar (excluding lactose and galactose) intake of children, the Anses has identified two levers: reduction in sweetened beverages (soft drinks and fruit juice) and reduction in pastries/biscuits/cakes. These foods, which are frequently offered as snacks, can be replaced by foods that have less total sugars. In line with Anses report, French High Council of Public Health (Haut Conseil de la Santé Publique; HCSP) recommended limiting the consumption of such sweet foods, especially those that are both high fat and high sugars (pastries, chocolate, dairy desserts and ice creams, and spreads) and promoting products without added sugars^(6)^.

It is necessary to generalise the consumption of fruits in all their forms in order to provide diversity and replace sweet and fatty foods, especially at snack time^(13)^. Fruit juices (100 % pure juice) count as a form of fruit, but up to a maximum of one serving (a glass of 150 mL) per d^(5)^ in light of their high free sugars content and their reduced fibre content. In this way, studies demonstrated the nutritional benefits of replacing fruits juices by fresh fruits in children’s diet^(14,15)^. Apples are popular fruit across France and Europe^(16)^, and children have preference for ready-to-eat food because they are easy to get and carry^(17)^. If fresh fruits must be encouraged, other forms like apple puree appears as a complement in order to reach the recommended five fruits and vegetables a day. An American study based on a representative sample of children between 2 and 18 years old demonstrated that consumers of apple in all their forms had significantly higher total intakes of dietary fibres, Mg, and potassium and lower intakes of added sugars than non-consumers^(18)^. In France, apples are also consumed by both adults and children in the form of puree. In 2022, a survey of 1000 adults representative of the French population showed that 91 % of households reported consuming fruit puree, with 60 % stating that they consume them at least once a month^(19)^. The consumers of processed fruits choose them because they can be eaten throughout the year, are convenient and have a longer shelf life, and 82 % of them even believe that processed fruits help increase their overall fruit consumption. The no-added sugar fruit puree is even included in public health recommendations as one serving of fruit in the recommended 5-a-day intake^(20)^. However, to the best of our knowledge, no study has focused on the addition of fruit in the form of puree, especially if it were to replace other foods that are sources of sugars and SFA at different times of the day including snacks.

The objective of the present study was to evaluate the nutritional impact of consuming a portion of fruit puree on four different times of consumption (breakfast, lunch, snack and dinner), with and without the substitution of other pre-defined sugary food and drinks. The simulation on children’s diet was performed, in a nationally representative sample of French children from 1 to 17 years old using INCA3 survey.

## Methods

The study was conducted in three stages on the basis of national French dietary data. First, sociodemographic characteristics and main nutritional intakes of consumers and non-consumers of all types of fruit purees were compared. Then, the introduction of no-added sugar fruit puree was simulated at different eating occasions to reach one serving. Finally, this introduction was evaluated as a substitution of other pre-defined food items. Simulations and analysis were performed on children from 1 to 17 years old, divided into five different age groups corresponding to INCA3 age ranges, that is, 0–3, 4–6, 7–10, 11–14 and 15–17 years old.

### Data

Dietary data on consumption in France came from INCA3 survey^(21)^. INCA3 is based on two or three non-consecutive 24-h dietary recalls (two weekdays and one week-end day). Only data for children (*n* 1993) were used. After removal of toddlers under 1-year-old, the final sample was composed by 1934. Gender and age of children as well as socio-economic characteristics and standard of living of his/her representative were provided in INCA3 data. As part of the dietary intake assessment, participants needed to name the eating occasion among ten possibilities. To simplify the analysis, occasions corresponding to ‘Before breakfast’, ‘Breakfast’, and ‘In the morning’ were grouped into Breakfast, ‘Aperitif before lunch’ and ‘Lunch’ were grouped into Lunch, ‘Snack’ and ‘In the afternoon (excluding snacks)’ were groups into Snacks, and ‘Aperitif before dinner’, ‘Dinner’, and ‘In the evening/night’ were grouped into Dinner.

Energy and nutrient values per 100 g of each food consumed were provided in the INCA3 database. Only free and added sugars were estimated by our research team following Louie et al procedure^(22)^. Each food item was classified according to INCA3 code, FOODEX 2 facets^(23)^ and GLOBODIET^(24)^ categories.

References values for macronutrients, vitamins and minerals were based on the most recent Anses dietary recommendations and on FAO/WHO scientific opinion for free sugars^(25)^. More specifically, vitamins and minerals daily recommended values came from the new dietary reference values for the vitamin and mineral intake of the French population updated in 2021^(26)^, proteins, carbohydrates, fibres and fats from the 2019 opinion on the updating of the French National Nutrition and Health Program dietary guidelines for children^(11,13)^, and SFA and unsaturated fatty acids from the 2011 updating of the recommended fatty acids intakes^(27)^. The more demanding recommended intake was taken into account where ages ranges for recommended values did not match INCA3 age ranges. Nutrient references values are summarised in online supplementary material, Supplemental Table S1.

### Determination of consumers and non-consumers of fruit puree

A consumer of fruit puree was defined as a child who consumed at least one time fruit puree (with or without added sugars) over the two or three recalls whatever the consumed amount. Ready-to-eat pureed fruits for children were considered as a fruit puree as well.

### Simulations

**Fig. 1:**
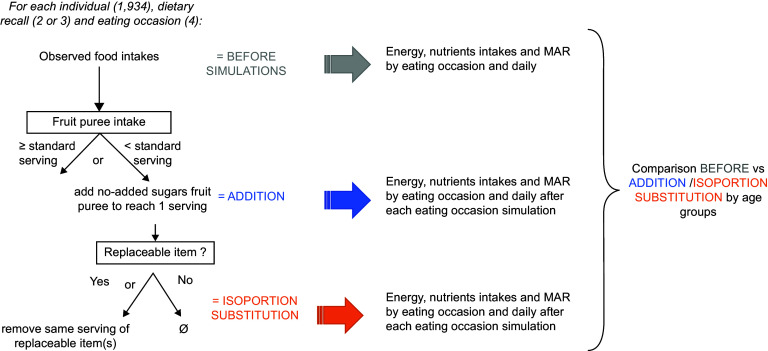
The principle of simulations and analysis. MAR, mean adequacy ratio

The introduction of no-added sugar fruit puree to reach one serving of fruit puree in French children’s diet was tested through each eating occasions (successively) and through two simulations: ‘ADDITION’ and ‘ISOPORTION SUBSTITUTION’. Simulations were performed each of the dietary recall considering each dietary recall independent from another (Fig. 1).

#### Standard fruit puree

The no-added sugar fruit puree introduced in the simulations was based on the nutritional composition of a ‘standard’ no-added sugars fruit puree (NASFP) by selecting the food label fruit puree associated with the foodex code ‘without added sugar’ from INCA3 database. The nutritional composition of the standard NASFP is detailed in online supplementary material, Supplemental Table S2.

Both simulations aimed to add or complete fruit puree intake to reach an amount corresponding to one serving. However, serving sizes may differ according to age and eating occasions. Standard serving sizes corresponded to the median NASFP portion consumed calculated by eating occasion and age groups in INCA3 recalls. The only exception was for children of 15–17 years old at snack time, where the serving size was set at 100 g (based on median portion size at other eating occasions) instead of an unrobust (because based on five recalls) estimation of 162 g. Standard servings of fruit puree are presented in online supplementary material, Supplemental Table S3. Usually, 90 g correspond to the serving size of a pouch, whereas 100 g is the serving size of a cup.

#### Replaceable items

The principle of the ISOPORTION SUBSTITUTION was to compensate the addition of one or less than one serving of no-added sugar fruit puree by the removal of the same serving of replaceable items, considered as high sugars and/or high fat. The list of replaceable items, without priority rank, was extracted from the list of sweet foods whose consumption need to be limited according to the 2020 opinion of the HCSP about the update of French dietary guidelines for children^(6)^. Replaceable items were (without ranking) soft drinks, fruits juices, pastries, biscuits, dairy desserts, ice cream and chocolate confectionary. They were identified thanks to GLOBODIET classification in INCA3. Standard servings of replaceable items were estimated in the same way as for fruit puree (see above) and are presented in online supplementary material, Supplemental Table S3.

#### Details of the simulations

For each eating occasion and each age group, two simulations were performed: ‘ADDITION’ and ‘ISOPORTION SUBSTITUTION’. Whatever the simulation, the participant had to consume less than one serving of fruit puree at the given dietary recall and given eating occasion to enter the simulation. The principles of both simulations are described in Fig. 1:

- NO-SUBSTITUTION: This simulation aimed to evaluate the direct effect of the introduction of a no-added sugar fruit puree among participants who consume little (less than one serving at the eating occasion) or no fruit puree. Thus, the simulation introduced the necessary amount to reach the serving of fruit puree without compensating for other foods.
- ISOPORTION SUBSTITUTION: The simulation was only performed on eating occasions where fruit puree has been introduced in ADDITION step and at least one replaceable item of the list (see above) is consumed. It compensated for the introduction of fruit puree by the removal of the same serving of replaceable food item(s). If several replaceable food items were consumed, they were removed in equal proportions. If the amount of replaceable item(s) was not high enough, it was entirely removed without modifying the amount of fruit puree introduced (so there may be a difference in portion or energy between before and after the substitution)

The simulations were conducted for each eating occasion successively, and not simultaneously, to accurately simulate only one eating occasion per d.

### Analysis

#### Comparison between consumers and non-consumers of fruit puree

Children in INCA3 were categorised into consumers or non-consumers according to the previous definition. The distribution of six qualitative variables reflecting individual socio-economic characteristics was estimated and compared between consumers and non-consumers of fruit puree. The qualitative variables included gender, age, socio-professional category (SCP) of the reference person for the household, total monthly income of the household per consumption unit, level of education of the reference person and food insecurity status of the reference persons.

The overall nutritional quality of the diets was assessed through the average mean adequacy ratio (MAR), an indicator that estimates the average content of essential nutrients expressed as a percentage of daily recommended intakes^(28)^. In the present study, the twenty-three nutrients taken into account in MAR calculation were proteins, fibres vitamins B_1_, B_2_, B_6_, B_9_, B_12_, C, D, E and A, Ca, potassium, Fe, Mg, Zn, Cu, iodine, Se, linoleic acid, α-linolenic acid and DHA. The ratio for each nutrient was truncated at 100 so that a high content of one nutrient could not compensate for the low intake of others as indicated in Equation (1):
(1)

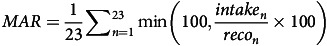

where 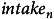 is the total daily or per eating occasion intake of *nutrient*_*n*_ and 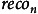 is the recommended daily intake of the same nutrient.

Average free sugars intakes and SFA both in percentage of EI and MAR were calculated among all ages and compared according to the status of fruit puree consumption. Average amounts of fruit puree consumed by eating occasion were also calculated. Fibres intakes were analysed according to age groups because needs in this nutrient differ from an age class to another.

#### Analysis of the simulations

Energy and nutrients intakes were estimated by calculating the average intakes over the three recalls daily (all eating occasions combined) and by eating occasion. Nutrients intakes were expressed in their reference units and as a percentage of the recommended daily intakes.

The analysis of the simulations consisted in comparing the nutritional quality of children diets before (observed diets) and after (modelled diets) each simulation (Fig. 1).

- By eating occasion: energy, free sugars, SFA and MAR were compared between observed intakes and both ADDITION and ISOPORTION SUBSTITUTION simulated intakes, for each eating occasion.
- Daily, after each simulated eating occasion: The percentage of children meeting the daily recommended nutrients intakes was compared between observed daily intakes and both ADDITION and ISOPORTION SUBSTITUTION simulated intakes.

#### Statistical analysis

Qualitative variables were compared between consumers and non-consumers using *χ* ^2^ tests. Quantitative variables were compared using *t* tests for paired measures to assess whether the variation in individual nutritional shifts between observed and simulated intakes was significantly different from zero. *χ* ^2^ tests were also applied to compare the percentage of children meeting the daily recommended nutrients intakes between observed and simulated intakes. To ensure sample representativeness, all analyses accounted for the INCA3 sampling frame design and were weighted for unequal sampling probabilities and differential non-responses by gender, age, size of the household, season, region, size of urban area and occupation and socio-professional category of the reference person for the household. A significance level of 5 % was applied. All analyses were conducted using R version 4.2^(29)^.

## Results

### Characteristics of consumers and non-consumers of fruit puree

Table 1 shows the sociodemographics characteristics of the INCA3 children according to their status of fruit puree consumption. Fruit puree consumption was associated with age with 78 % of consumers being less than 10 years old, while this age group represented 59 % of the total sample. Teenagers (11–17 years old) who represented 40·9 % of the total sample were overrepresented in the non-consumers subsample with 57·2%. High socio-professional category and high level of education were overrepresented among consumers (28 % and 23·5 %, respectively) compared with non-consumers (19·3 % and 15·4 %, respectively). Consumers were less likely to be part of a low income per consumption unit household (19·3 %) or in food insecurity status (5·65 %) than non-consumers (35·2 % and 14·3 %).

**Table 1:** Sociodemographic characteristics of consumers and non-consumers of fruit puree (all age ranks combined)

		All	Consumers	Non-consumers	*P*-value
%		*n* 1934	45·9	54·1	
Gender	Men	51·3	49·4	53	0·327
	Women	48·7	50·6	47	
Age	1–3 years old	17·0	26·1	9·34	<0·001
	4–6 years old	18·3	25·5	12·3	
	7–10 years old	23·7	26·7	21·2	
	11–14 years old	24·0	15·3	31·4	
	15–17 years old	16·9	6·46	25·8	
Socio-professional category	Low	39·6	36·4	42·4	<0·001
	Medium	31·3	32·7	30·1	
	High	23·3	28	19·3	
	Not working	5·51	2·41	8·15	
	Don’t know/refuse to answer	0·26	0·47	0·08	
Income per consumption unit (CU)	<900 €/month/CU	27·9	19·3	35·2	<0·001
	(900–1340) €/month/CU	27·4	29·5	25·6	
	(1340–1850) €/month/CU	25·2	27·8	23	
	>=1850 €/month/CU	12·7	15·9	10	
	Not specified	6·81	7·53	6·2	
Level of education	Primary + middle school	43·5	38·2	48	<0·001
	High school	17·8	16·5	18·9	
	1–3 years of post-secondary education	18·1	21·3	15·4	
	4 or more years of post-secondary education	19·0	23·5	15·1	
	Not specified	1·65	0·50	2·63	
Food insecurity level	High	2·97	0·95	4·69	<0·001
	Moderate	7·36	4·70	9·63	
	Security	89·7	94·4	85·7	

Table 2 shows fresh fruits and fruits juices consumption as well as main nutrients intakes according to children’s status of fruit puree consumption. Although non-consumers were older than consumers, they did not consume significantly more fresh fruits. However, non-consumers consumed on average 20 g more fruits juices than consumers. Added sugars and Na that should be limited were also higher among non-consumers, but SFA percentage remains the same. Added sugars intakes were, respectively, 11·8 % and 12·8 % in consumers and non-consumers. Intakes in fibres were either non-significantly different between the two groups or higher in consumers group compared with non-consumer one.

**Table 2:** Main nutrient intakes of consumers and non-consumers of fruit puree (all age ranks combined except for fibres)

	Ages	All	Consumers	Non-consumers	*P*-value
Fresh fruits intakes (g/d)	All	73·9	70·6	76·7	0·234
Fruit juice intakes (g/d)	All	89·3	78·7	98·3	0·009
MAR* (%)	All	84·7	87·4	82·4	<0·001
Fibres (g/d)	1–3	11·3	11·5	10·8	0·324
	4–6	13·5	14·2	12·2	<0·001
	7–10	15·6	16·4	14·8	0·006
	11–14	17·7	17. 7	17·8	0·948
	15–17	16·2	18·5	15·8	0·004
Added sugars (% EI)	All	12·3	11·8	12·8	0·001
Free sugars (% EI)	All	13·7	13·0	14·2	<0·001
SFA† (% EI)	All	14·5	14·5	14·5	0·984

MAR, mean adequacy ratio; EI, energy intake.

* Mean adequacy ratio.

† SFA.

**Fig. 2:**
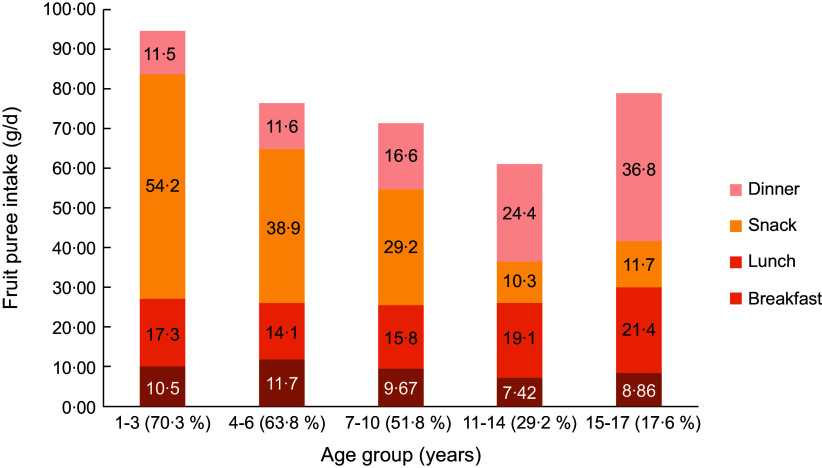
Average amount of fruit puree consumed per eating occasion and age among consumers of fruit puree

Figure 2 displays the average amount of fruit puree consumed in the different age groups among consumers of fruit puree in the whole day. Total amounts were split per eating occasion for each age group, and *X* labels show the prevalence of consumers in each age group. Prevalence of fruit puree consumption decreased with age from more than 60 % of consumers in the two youngest groups to less than 20 % in the oldest one. Among those consumers, average amount of fruit puree consumed decreased from 1–3 years old (95 g/d on average, corresponding to 5 g more than the size of a pouch) to 11–14 years old (60 g/d), mainly explained by a strong decrease at snack time, while consumptions at lunch and dinner went slightly up. Then, total amount consumed goes up in 15–17 years old to reach 80 g/d with almost an half consumed at dinner.

### Simulations

**Fig. 3:**
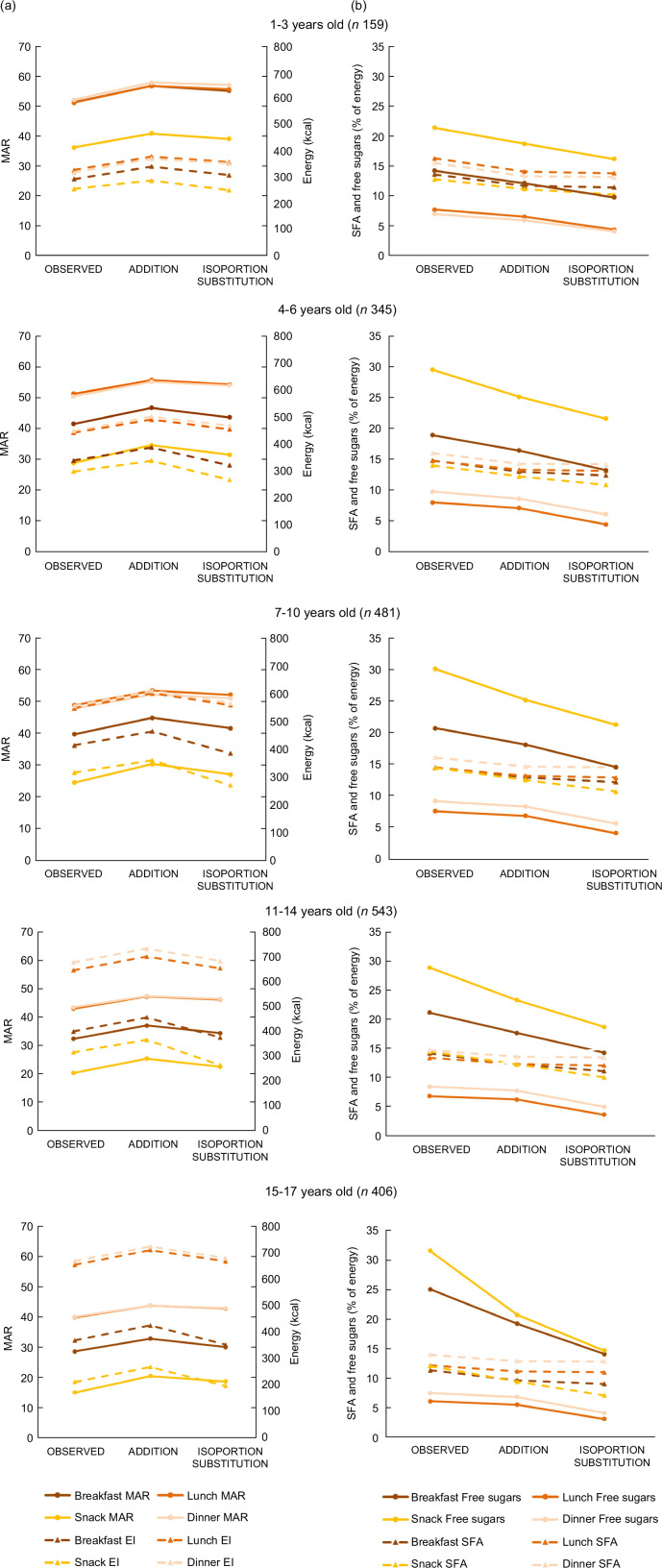
(a) Energy and MAR intakes estimated at eating occasion in observed and simulated diets. (b) SFA and free sugars intakes estimated at eating occasion in observed and simulated diets. All differences between observed diets and simulated diets by occasions were significant. MAR, mean adequacy ratio; EI, energy intake

Whatever the eating occasion, introducing fruit puree and substituting it with a reduction of replaceable food items improved the MAR of children diet (Fig. 3(a), full line). First, with the addition of no-added sugar fruit puree only (ADDITION), that brought about 50 kcal (Fig. 3(a), dash line) combined to essential nutrients like fibres, MAR was increased. Then, the substitution (ISOPORTION SUBSTITUTION) maintained MAR closed to the ADDITION level while reducing the energetic intake thanks to the removal of empty calories from replaceable food items. It was especially visible for oldest age groups which were less likely to consume fruit puree in their observed diet and, as a consequence, more likely to benefit from simulations. For example, for 15–17 years old, almost all (98·8 %) of observed snacks benefits from the simulation (see online supplementary material, Supplemental Fig. S4) leading to an increase in MAR by 24% for a similar EI (–6 %) between observed and ISOPORTION SUBSTITUTION.

Simulations had a beneficial impact on both SFA and free sugars intakes (Fig. 3(b)) first because introducing fruit puree in the diet increase EI without adding SFA and free sugar, leading to a decrease in their percentage of energy. Then, the decrease in free sugars was even greater in ISOPORTION SUBSTITUTION at breakfast and snack thanks to the decrease of replaceable food items which have high sugar contents. For example, for children between 7 and 10 years old, compared with observed diets, SFA decreased respectively from 1·6 points and 2·3 points and free sugars decreased from respectively 2·6 and 6·2 points after ADDITION and ISOPORTION at breakfast.

**Fig. 4:**
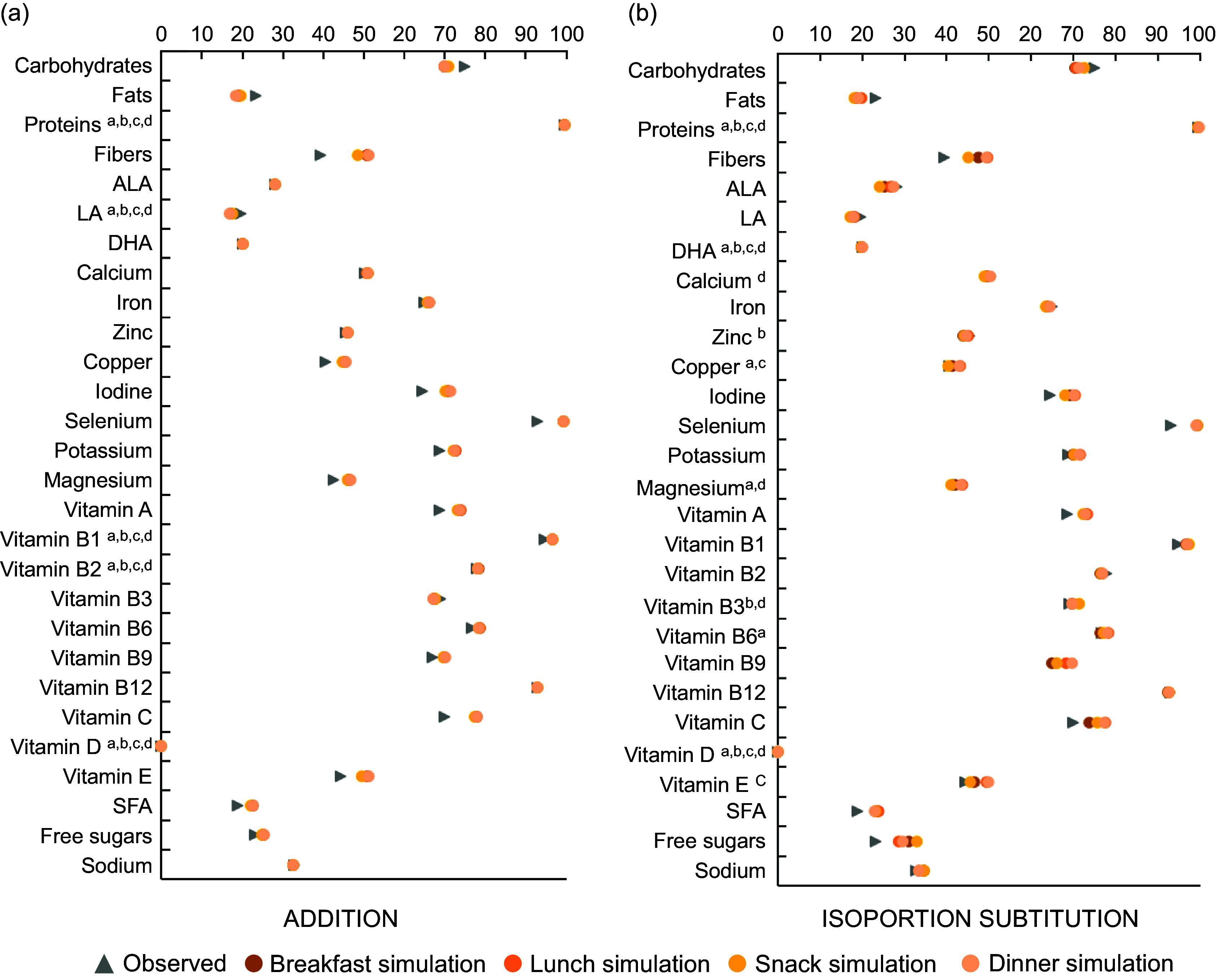
Prevalence of children fulfilling nutritional requirements before and after (a) ADDITION and (b) ISOPORTION SUBSTITUTION, all age groups mixed (1–17 years old). All *χ* ^2^ tests between observed and simulated content were significant except for the nutrients indexed. ^a^Non-significative (ns) at breakfast, ^b^ns at lunch, ^c^ns at snack, and ^d^ns at dinner. ALA, α-linolenic acid; LA, linoleic acid

Figure 4 displays the prevalence of children with adequate intake for all nutrients before and after eating occasions-specific simulations, for all age groups combined. The largest positive impacts of both simulations were observed for fibres, iodine, Se, vitamin A, vitamin C, SFA and free sugars, while prevalence of children with adequate carbohydrates intake was slightly degraded. More precisely, introducing fruit puree only in ADDITION increased the percentage of children meeting recommendations for fibres, iodine and vitamin C (+11, +6·5 and +7·8 points in the prevalence of adequate intake, respectively), but the substitution step in ISOPORTION SUBSTITUTION results in slightest increase. For Se, the benefit of about +6 points was equivalent between the ADDITION and the ISOPORTION SUBSTITUTION simulations. For SFA and free sugars, the benefit coming from fruit puree introduction was amplified by the substitution (+4·4 and 7·4 points, respectively). The trend is the same for all age groups, except that the observed prevalence of teenagers is lower than children (see online supplementary material, Supplemental Tables S5).

## Discussion

Our results showed that the promotion of a portion of NASFP consumption at different moments of consumption may improve nutritional quality of children’s diet, especially those of oldest children who are currently low consumers of fruit puree and fresh fruits^(9)^. When fruits puree were consumed as substitute of sweetened and/or fatty products, nutritional benefits were multiplied by maintaining improvement of favourable nutrients intakes brought by fruit puree while reducing intakes of unfavourable ones brought by replaceable foods. Best improvement in nutrient intakes were observed for fibres and free sugars, with respective increases of 17 points for 4–6 years old (see online supplementary material, Supplemental Table S5A) and 10·5 points (see online supplementary material, Supplemental Table S5B) for 15–17 years old in their prevalence of adequacy. Increase in fibre intakes is all the more an important point as the present percentage of French children meeting fibres recommendations is low, especially in the oldest group (15–17 years old) where it reached only 20·7 % (see online supplementary material, Supplemental Fig. S5). While the best benefits on favourable nutrients such as fibre were observed after fruit puree introduction alone, free sugars intake reduction was mainly attributed to the reduction in sweetened/fatty products, especially at snack and breakfast, demonstrating the benefit of following recommendations of the French public health agencies to reduce added sugars.

Our analysis allows to differentiate the impact of introducing no-added sugar fruit puree from the one of reducing sweetened/fatty products on diets’ nutrient content. First, the addition of no-added sugar fruit puree allows an increase of favourable nutrients into diets (as shown by MAR increases) associated with a modest increase (from 32 to 57 kcal) in EI. The nutrient density (high content in nutrients to favour associated with a low energy content) of fruits and vegetables has already been described by the past and its health benefit is acknowledged widely^(30)^. The reduction of sweetened/fatty products lead to a decrease in energetic intake (from –18 to –101 kcal compared with the introduction alone) with associated unfavourable nutrients, without impacting strongly the content in favourable ones. The need for obesity prevention to decrease consumption of empty or discretionary calories foods has also been promoted by the past and is largely acknowledged^(31)^. The proposed simulation is thus in accordance with the principles of public health strategies aiming to promote nutrient dense foods while limiting energy-dense ones^(32)^. The prevalence of overweight and obesity among children in France is at high levels: it reached, respectively, 29 % and 17 % for 2–4-year-old children, 39 % and 19 % for 5–7-year-olds, and 29 % and 8 % for 8–10-year-olds in September 2020^(33)^. According to the school surveys^(34)^, it would have even significantly increased compared with the previous years, marking an acceleration in growth since the global health crisis. No direct link between fruit and vegetables consumption and childhood BMI has yet been clearly established^(35)^. However, combined with decreased intakes of high-fat/high-sugar foods, increased intakes of fruits and vegetables could prevent obesity in families at risk^(36,37)^. Our results suggested that, in the same way as fruits and vegetables, the substitution of foods of lower nutritional quality by no-added sugars fruits pureed would helped to limit the EI of meals. In complement to fruits and vegetables, no-added sugar fruit purees would not compromise the associated benefits in terms of calories and could thus participate in the prevention of childhood overweight and obesity.

According to our results, identified barriers to break down in order to shift towards an increased consumption of fruit puree were their accessibility for low socio-economic status as well as their attractivity for oldest children. The link between fruits and vegetables consumption and sociodemographic features is well established in the literature^(8,9,38,39)^. In France, children were more likely to be low consumers of fruits and vegetables as their parents’ education level decreased, and high levels of sugar-sweetened beverage consumption were more common among children whose parents had less than 3 years of education^(8)^. Similarly, the proportion of children who did not consume vegetables the 3 d of the third national survey was higher among children of workers than among children of managers^(9)^. The level of fruit puree and fruits in syrups consumption also increase with their representative education level. Fruit puree consumption has not been evaluated alone. To the best of our knowledges, the present study is the first to analyse the place of fruit puree alone in children’s diet and to compare its consumptions according to socio-economics status. It has been shown that, as consumers of other form of fruits, consumers of fruit puree were more likely to be part of families with high economic status than non-consumers. Yet fruit puree is cheaper than many sweet products such as chocolate and cookies, suggesting that, on the contrary to fresh fruits and vegetables^(40)^, their price is not an obstacle to their consumption. Moreover, apart from cost, availability and accessibility as well as taste preferences drive the consumption of fruit and vegetables among children^(41)^. In this way, fruit puree could expose children to fruits taste and thus facilitate their consumption. However, further investigations are needed to identify the best strategies to increase the prevalence of compote consumption among French children, especially those from socio-economically disadvantaged families. The present study showed that adolescents were less likely to consume fruit puree than young children and that a switch from snack to others eating occasions was observed with increasing age, leading to a better benefit of simulations at this age and eating occasion. The reduction of the mid-afternoon snack eating occasion attractivity with increasing age has already been described in the INCA3 report^(9)^ which shows that contribution of snack to daily consumption (in g) is 13·9 % in 0–10 years old children while it falls to 8·2 % in 11–17 years old. In accordance with the present study, the INCA3 report also describes the switch of fruit puree and fruits in syrup consumption from snack to others eating occasions (mainly lunch and dinner) with increasing age. Indeed, among consumers only, 48·4 % of daily amount of fruit puree and fruits in syrup were consumed at snack in youngest children (0–10 years old) against 16·3 % in adolescents (11–17 years old). Vigilance must be paid on fruits intakes in adolescents because the reduction in fruit puree and fruits in syrups consumption with increasing age is not compensated by an increase of fresh fruits consumption while fruits juice consumption slightly increase.

Moreover, studies have shown that children and adolescents are the main consumers of ultra-processed foods^(42)^. In France, the analysis of INCA3 data (1–10 years; *n* 1035) evaluates at 45·5 % the share of calories provided by ultra-processed foods in 2014–2015, constantly increasing with time compared with 42·8 % in INCA1 survey 1998–1999 and 43·2 % in INCA2 survey 2006–2007^(43)^. In addition to nutritional deficiencies, some studies suggest a negative impact of high ultra-processed consumption on the academic abilities of children and adolescents^(44)^, an increase in body fat index and BMI, dyslipidemias and metabolic syndrome^(45,46)^. In the present study, the list of substituted items (soft drinks, fruit juices, pastries, cookies, dairy desserts, ice cream and chocolate confectionary) is dominated by ultra-processed food (except for 100 % pure fruit juice), so it would be all the more beneficial for the children’s health if they were replaced by a little processed food that is the NASFP.

With a quarter of greenhouse gas emissions coming from food production^(47)^, current researches on dietary shifts to promote need not only to assess nutritional consequences of proposed shifts but also their environmental consequences. Processed foods have a longer life cycle than unprocessed ones, conducting to higher impacts per kg. As an example, 1 kg of fruit puree has an impact on climate change of 0·845 kg CO2 against 0·397 kgCO2 for raw apple because of processing and packaging steps^(48)^. However, processed foods may play a role in the reduction of food waste. Indeed, with about 41 % of fruits available in the EU that is wasted^(49)^, mainly due to their fragility, processed fruit, by keeping quality and extend the shelf life of fruit for as long as possible, may play a role in the attainment of the 12·3 reduction of food waste and losses Sustainable Development Goals (‘By 2030, halve per capita global food waste at the retail and consumer levels and reduce food losses along production and supply chains, including post-harvest losses’). Quantitative impacts of a one a day fruit puree on the environment need to be done to assess its impact on environmental dimension.

This study has limitations that need to be acknowledged. First, for all children, specific eating occasions simulations were performed independently of what is consumed at others eating occasions. As a consequence, results express at a daily level (Fig. 4) may contain simulated diets that have more than one fruit puree a day. Indeed, between 5·75 % (for 15–17 years old at dinner) and 55·7 % (for 1–3 years old at lunch) of the simulations were performed on a recall where a fruit puree was already consumed on one of the three other eating occasions (results not shown). Moreover, only the amount of fruit puree consumed was considered to perform or not a simulation, meaning that other form of fruits consumed at the eating occasion were not taken into account. The amount of fresh fruits was not taken into account to enter or not the simulation. Therefore, adding fruit puree might have occurred for some children who already consume a portion (or more) of fresh fruit. Replacing a serving of fresh fruit with a serving of fruit puree would contradict public health recommendations, so not performing the simulation on eating occasions with already a serving of fresh fruit could have been an option. However, the objective was to simulate the impact of consuming fruit puree in addition to fruits, on all children, not just those whose consumption of fruits was insufficient. Likewise, replaceable items were not always available for substitution (see online supplementary material, Supplemental Fig. S4) leading to increases in energy content of simulated diets compared with observed ones in some eating occasions. Adapting for each child the best eating occasion to introduce fruit puree in the diet would be an interesting method but would stand on hypothesis difficult to defend. Third, simulation was performed where the consumption of fruit puree was insufficient, whether it contains added sugars or not. Thus, fruit puree with added sugars could have been completed with NASFP instead of being totally removed. However, the study is intended to remain theoretical and demonstrate the nutritional benefits of fruits puree that should be adapted to real conditions as complement of fruit and vegetables intakes.

Simulated diets with addition of NASFP were more nutrient-dense thanks to an increase of favourable nutrients (especially fibres) from no-added sugar fruit puree and a decrease in energetic intakes and free sugars from the substitution step. This study supports public health messages to children by quantifying the nutritional benefits of substituting sugary products by a healthier alternative, especially at breakfast and snack time.

## Supporting information

Poinsot et al. supplementary material Poinsot et al. supplementary material

## Supplementary material

For supplementary material accompanying this paper visit https://doi.org/10.1017/S1368980024000739
