# Supplementary material for: Nutritional impact of no-added sugar fruit puree consumption at different eating occasions: a modelling study on French children
Source: Public Health Nutr. 2024 Mar 26;27(1):e103. doi: 10.1017/S1368980024000739 (PMC11010159; doi:10.1017/S1368980024000739)
Supplement: Poinsot et al. supplementary material [file S1368980024000739sup001.docx]

## Supplemental Table S1 References values for macronutrients, vitamins and minerals

| **Nutrient** | **Unit** | **Minimum Daily Recommended Intake** | | | | |
| --- | --- | --- | --- | --- | --- | --- |
|  |  | **1-3 yo** | **4-6 yo** | **7-10 yo** | **11-14 yo** | **15-17 yo** |
| Carbohydrates | % EI | 40-50 | 40-55 | 40-55 | 40-55 | 40-55 |
| Fats | % EI | 45-50 | 35-40 | 35-40 | 35-40 | 35-40 |
| Proteins | % EI | 6 | 6 | 7 | 9 | 10 |
| Fibers | g | 10 | 14 | 16 | 19 | 21 |
| LA | % EI | 2.7 | 4 | 4 | 4 | 4 |
| ALA | % EI | 0.45 | 1 | 1 | 1 | 1 |
| DHA | mg | 70 | 125 | 125 | 250 | 250 |
| Calcium | mg | 450 | 800 | 800 | 1150 | 1150 |
| Iron | mg | 5 | 4 | 6 | Boys : 11  Girls : 13 | Boys : 11  Girls : 13 |
| Zinc | mg | 4.3 | 5.5 | 7.4 | 10.7 | Boys : 14.2  Girls : 11.9 |
| Copper | mg | 0.8 | 1 | 1.2 | 1.3 | Boys : 1.5  Girls :1.1 |
| Iodin | µg | 90 | 90 | 90 | 120 | 130 |
| Selenium | µg | 15 | 20 | 35 | 55 | 70 |
| Magnesium | mg | 180 | 210 | 240 | 265 | Boys : 295  Girls : 225 |
| Potassium | mg | 800 | 1100 | 1800 | 2700 | 3500 |
| Phosphorus | mg | 250 | 440 | 440 | 640 | 640 |
| Vitamin A | RE | 250 | 300 | 400 | 600 | Boys : 750  Girls : 650 |
| Vitamin B1 | mg/MJ | 0.0 | 0.072 | 0.072 | 0.072 | 0.72 |
| Vitamin B2 | mg | 0.6 | 0.7 | 1 | 1.4 | 1.6 |
| Vitamin B3 | mg/MJ | 1.3 | 1.3 | 1.3 | 1.3 | 1.3 |
| Vitamin B5 | mg | 4 | 4.5 | 5 | 6 | 6 |
| Vitamin B5 | mg | 4 | 4.5 | 5 | 6 | 5 |
| Vitamin B6 | mg | 0.6 | 0.7 | 1 | 1.4 | 1.7 |
| Vitamin B6 | mg | 0.6 | 0.7 | 1 | 1.5 | 1.6 |
| Vitamin B9 | µg | 120 | 140 | 200 | 270 | 330 |
| Vitamin B12 | µg | 1.5 | 1.5 | 1.5 | 2.5 | 2.5 |
| Vitamin C | mg | 20 | 30 | 45 | 70 | 100 |
| Vitamin D | µg | 15 | 15 | 15 | 15 | 15 |
| Vitamin E | mg | 7 | 7 | 9 | 10 | Boys : 10  Girls : 8 |
| **Nutrient** | **Unit** | **Maximum Daily Recommended Intake** | | | | |
| Free sugars | % EI | 10 | 10 | 10 | 10 | 10 |
| SFAs | % EI | 12 | 12 | 12 | 12 | 12 |
| Sodium | mg | 800 | 1000 | 1200 | 1200 | 1500 |

Supplemental Table S2. Nutritional composition per 100 g of the no-added sugar fruit puree added in the simulation (INCA3^1^)

| Energy (kcal) | 57.4 | Vitamin A (RE eq.) | 59,2 | Magnesium (mg) | 6.10 |
| --- | --- | --- | --- | --- | --- |
| Proteins (g) | 0.43 | Vitamin B1 (µg) | 0.07 | Potassium (mg) | 140 |
| Carbohydrates (g) | 12.8 | Vitamin B2 (mg) | 0.01 | Calcium (mg) | 8.1 |
| Sugars (g) | 11.6 | Vitamin B3 (mg) | 0.24 | Iron (mg) | 0.14 |
| Free and added sugars (g) | 0.00 | Vitamin B6 (mg) | 0.033 | Cupper (mg) | 0.05 |
| Fibers (g) | 1.68 | Vitamin B9 (µg) | 7.48 | Zinc (mg) | 0.025 |
| Fats (g) | 0.34 | Vitamin C (mg) | 12.2 | Selenium (µg) | 25 |
| SFA (g) | 0.082 | Vitamin E (mg) | 0.66 | Iodine (µg) | 10 |
| Sodium (mg) | 2.38 |  | | | |

^1^French Agency for Food Environmental and Occupational Health & Safety (Anses) (2017) Opinion of the French Agency for Food, Environmental and Occupational Health & Safety on "the Third Individual and National Survey on Food Consumption (INCA3 survey). Maison-Alfort, France

Supplemental Table S3 Standard servings per age and eating occasion for the fruit puree added in the simulation and the replaceable items

| Age rank | Eating occasion | Standard serving (g) | | | |
| --- | --- | --- | --- | --- | --- |
|  |  | Fruit puree | Soft drinks | 100% fruit juice | Pastries |
| 1-3 years old | Breakfast | 90 | 152 | 104 | 30 |
|  | Lunch | 100 | 121 | 146 | 46 |
|  | Snack | 90 | 147 | 135 | 30 |
|  | Dinner | 100 | 139 | 78.0 | 36 |
| 4-6 years old | Breakfast | 90 | 126 | 146 | 40 |
|  | Lunch | 90 | 150 | 125 | 55 |
|  | Snack | 90 | 161 | 208 | 42 |
|  | Dinner | 100 | 149 | 156 | 50 |
| 7-10 years old | Breakfast | 90 | 152 | 146 | 50 |
|  | Lunch | 100 | 192 | 125 | 68 |
|  | Snack | 90 | 200 | 208 | 50 |
|  | Dinner | 100 | 192 | 166 | 79 |
| 11-14 years old | Breakfast | 100 | 200 | 156 | 63 |
|  | Lunch | 100 | 228 | 156 | 81 |
|  | Snack | 90 | 207 | 208 | 64 |
|  | Dinner | 100 | 224 | 149 | 87 |
| 15-17 years old | Breakfast | 100 | 250 | 218 | 65 |
|  | Lunch | 100 | 264 | 125 | 90 |
|  | Snack | 100 | 244 | 218 | 70 |
|  | Dinner | 100 | 264 | 189 | 90 |
| Age rank | Eating occasion | Standard serving (g) | | | |
|  |  | Biscuits | Dairy desserts | Ice Cream | Chocolate confectionery |
| 1-3 years old | Breakfast | 13 | 100 |  | 9.00 |
|  | Lunch | 10 | 100 | 40.2 | 10.0 |
|  | Snack | 14 | 100 | 39.1 | 12.0 |
|  | Dinner | 18 | 100 | 36.5 | 7.25 |
| 4-6 years old | Breakfast | 26 | 108 |  | 13.9 |
|  | Lunch | 18 | 100 | 40.2 | 10.0 |
|  | Snack | 28 | 89.8 | 43.5 | 16.4 |
|  | Dinner | 29 | 100 | 40.2 | 13.0 |
| 7-10 years old | Breakfast | 35 | 85.0 | 25.6 | 20.4 |
|  | Lunch | 20 | 100 | 44.2 | 10.8 |
|  | Snack | 35 | 100 | 52.2 | 19.0 |
|  | Dinner | 20 | 100 | 44.2 | 13.0 |
| 11-14years old | Breakfast | 40 | 125 |  | 22.0 |
|  | Lunch | 18 | 100 | 52.2 | 14.5 |
|  | Snack | 39 | 100 | 70.0 | 21.0 |
|  | Dinner | 24 | 100 | 59.6 | 17.5 |
| 15-17 years old | Breakfast | 42 | 100 |  | 20.7 |
|  | Lunch | 23 | 100 | 62.3 | 16.5 |
|  | Snack | 40 | 125 | 66.7 | 24.0 |
|  | Dinner | 32 | 100 | 64.3 | 19.5 |

Supplemental Figure S4 Different kind of simulations among all the studied recalls. No simulations : the serving size of fruit puree was already reached. No substitution : No replaceable items, only addition. Partial substitution : the quantity of fruit puree was greater than 0 but lower than the serving size. Full substitution : the quantity of fruit puree was equal to 0.

| 1-3 years old | 4-6 years old |
| --- | --- |
| 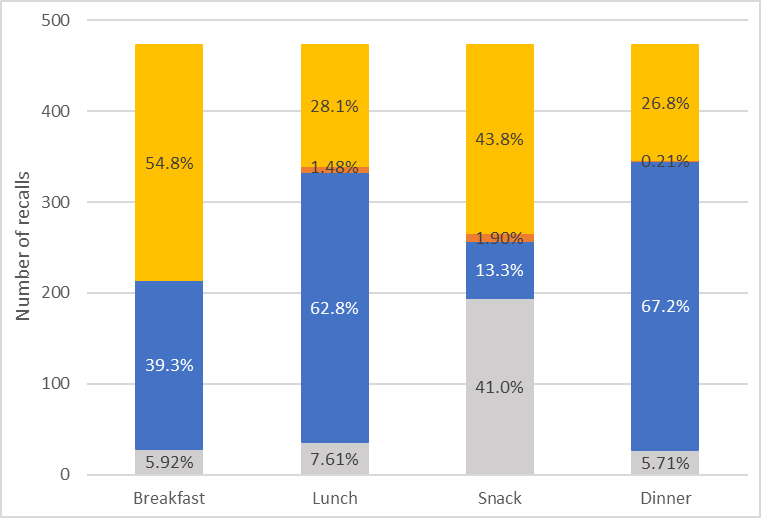 | 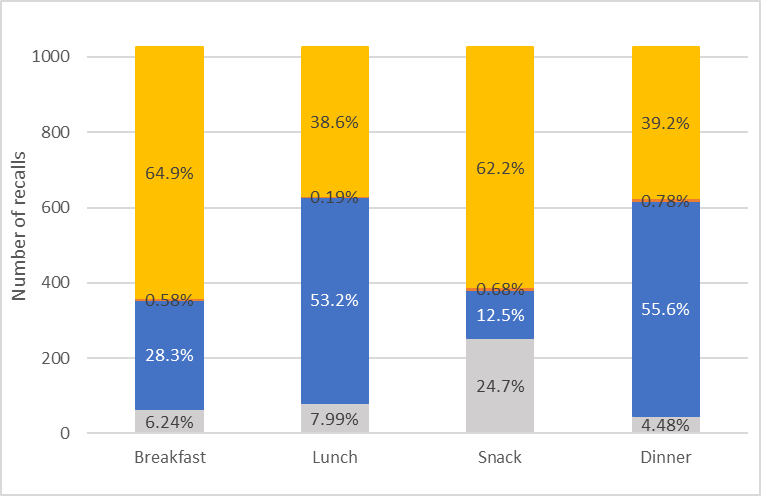 |
| 7-10 years old | 11-14 years old |
| 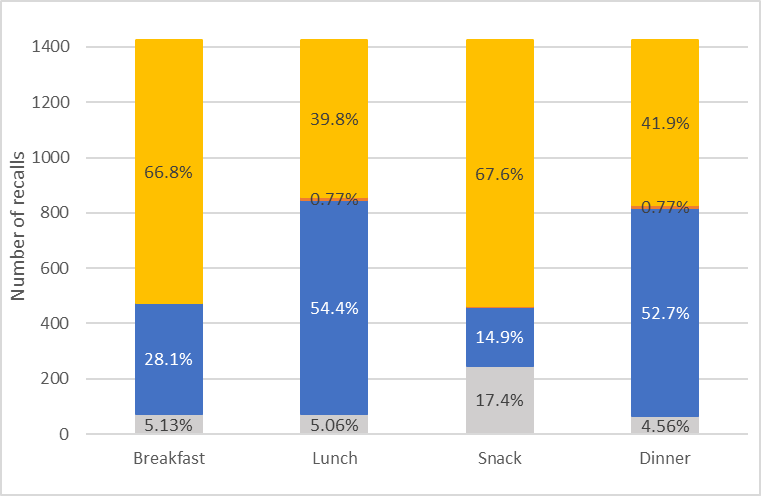 | 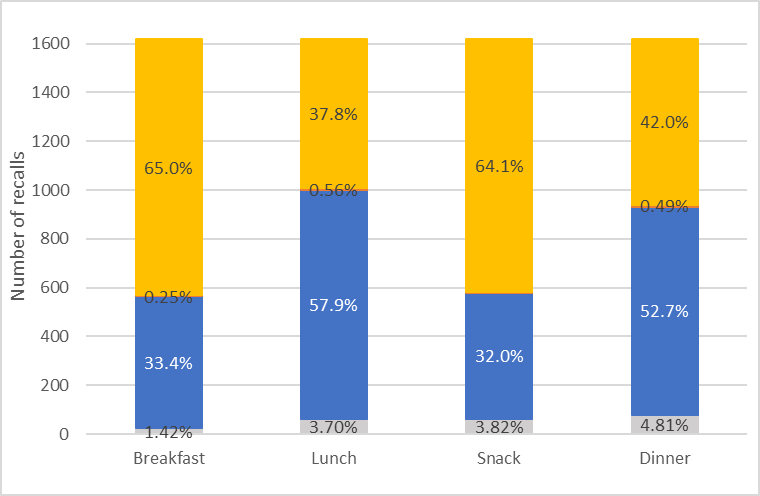 |
| 15-17 years old |  |
| 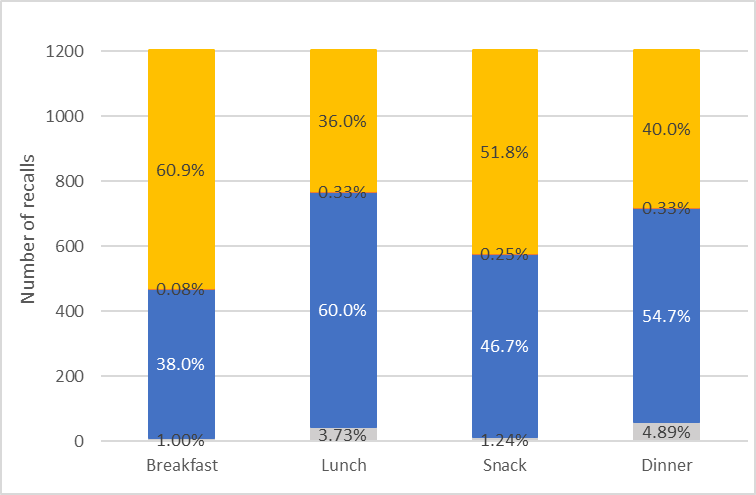 | 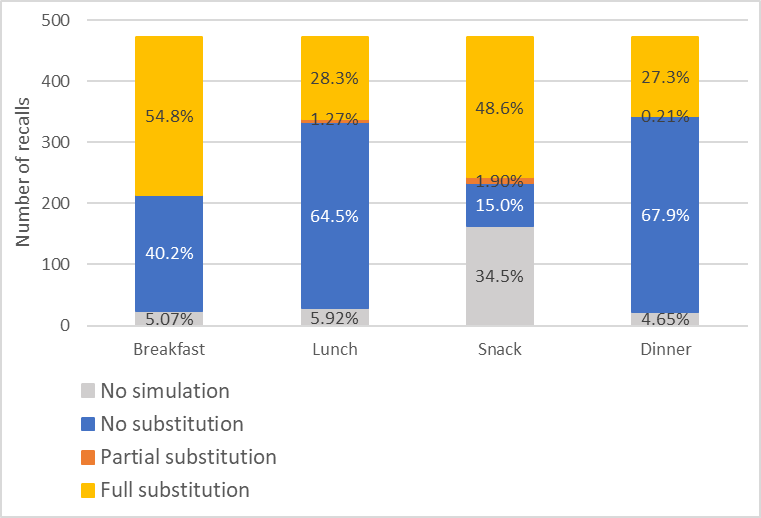 |

**Supplemental Figure S5A** Percentage of consumers fulfilling nutritional requirements (per age rank and for all age ranks combined) before and after ADDITION. detailed per age rank

| 1-3 (n=159) | 4-6 (n=345) |
| --- | --- |
| 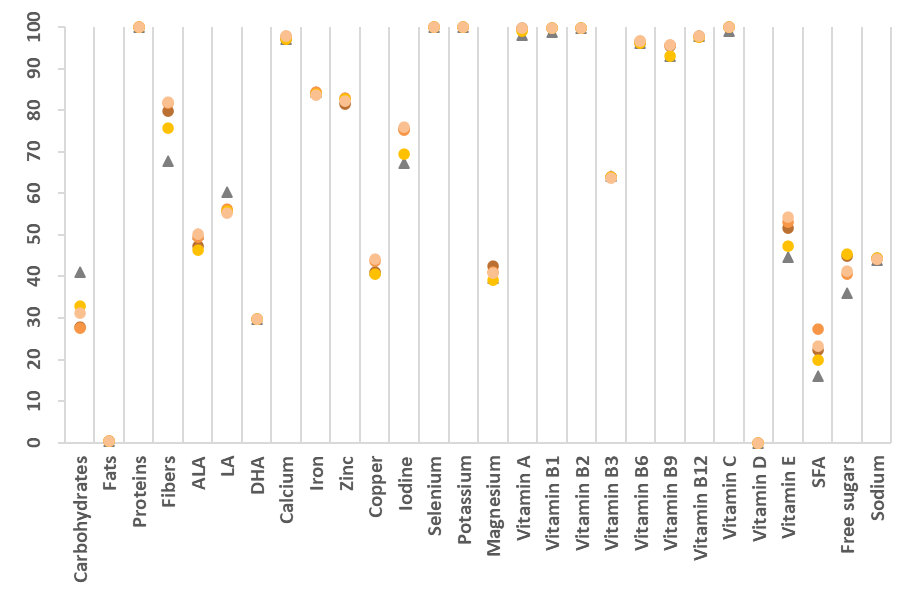 | 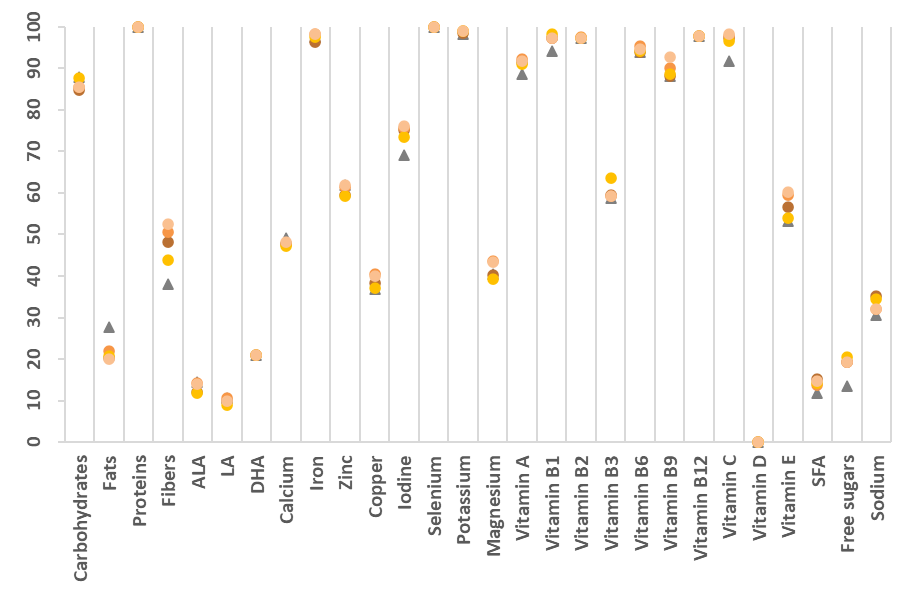 |
| 7-10 (n=481) | 11-17 (n=543) |
| 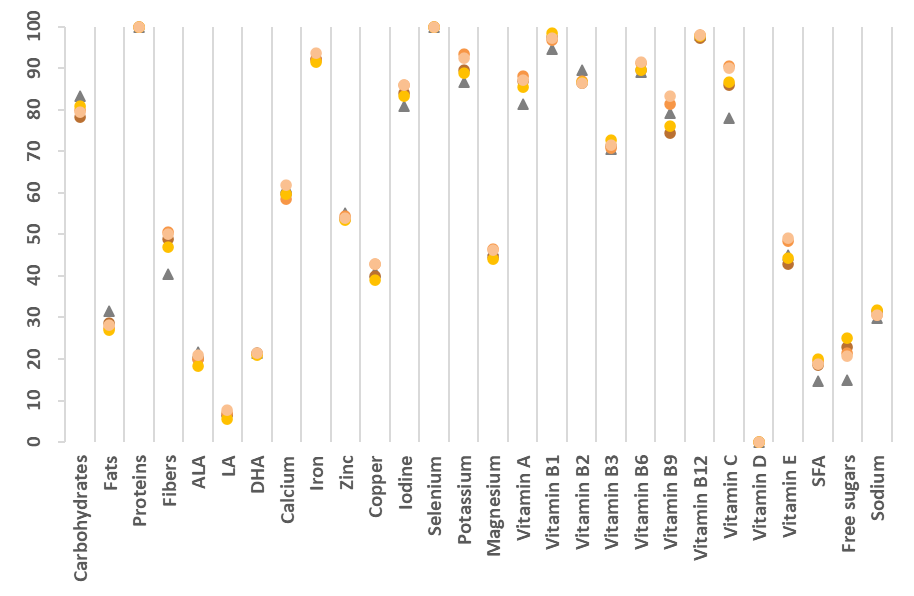 | 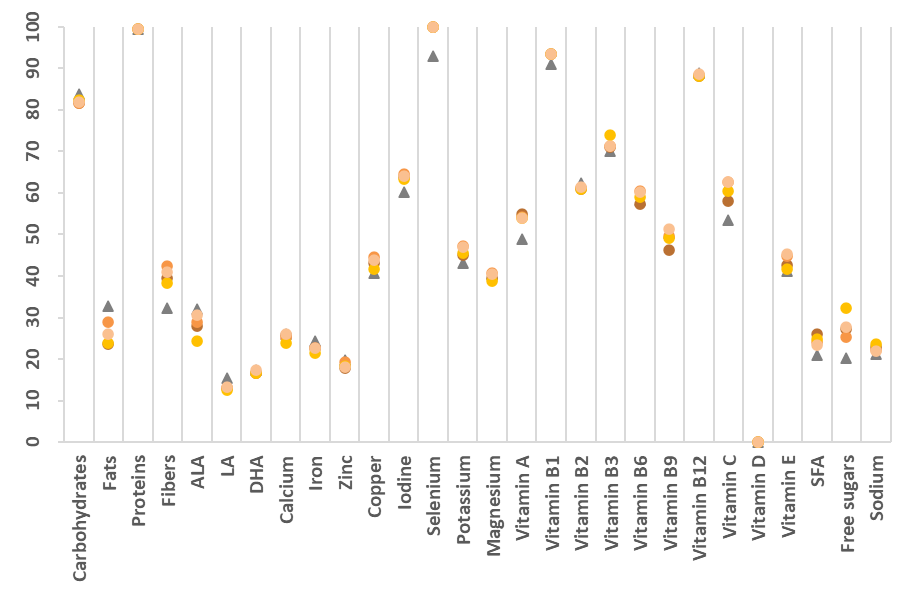 |
| 15-17 (n=406) |  |
| 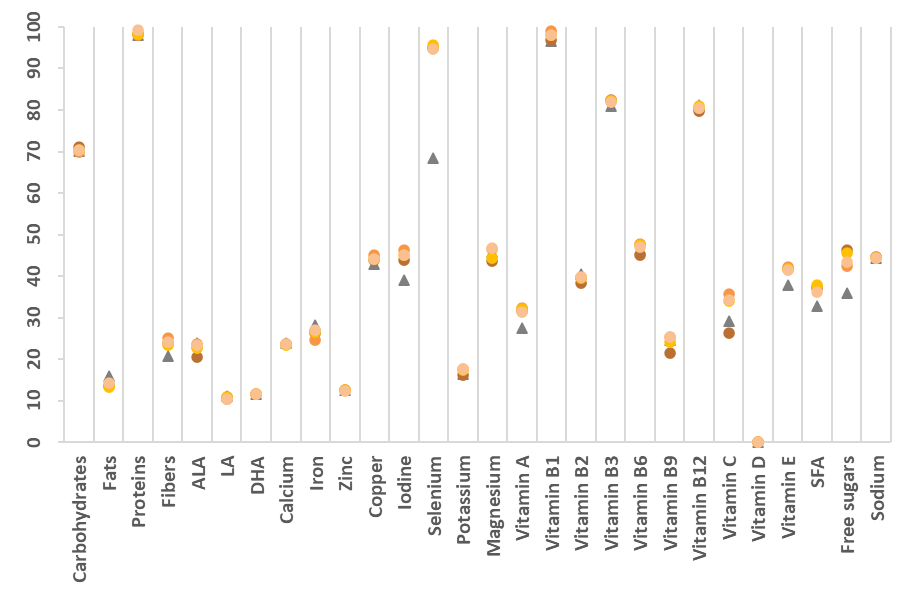 | 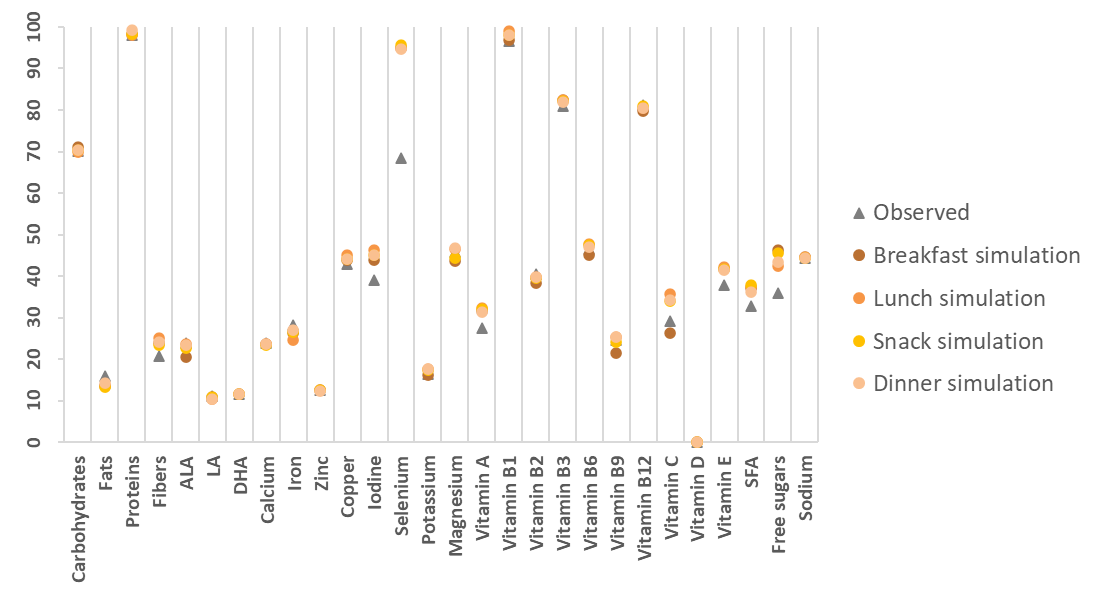 |

**Supplemental Figure S5B** Percentage of consumers fulfilling nutritional requirements (per age rank and for all age ranks combined) before and after ISOPORTION SUBSTITUTION. detailed per age rank

| 1-3 (n=159) | 4-6 (n=345) |
| --- | --- |
| 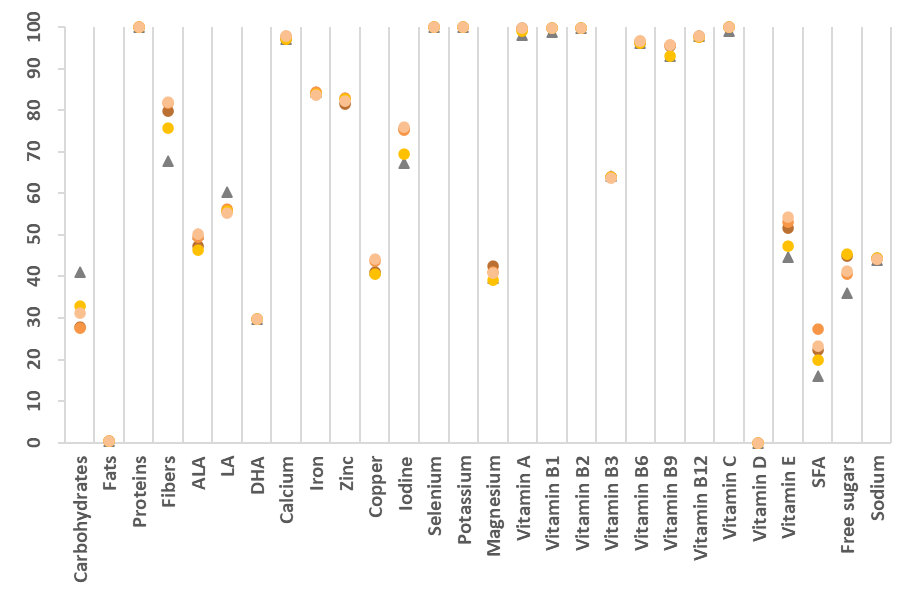 | 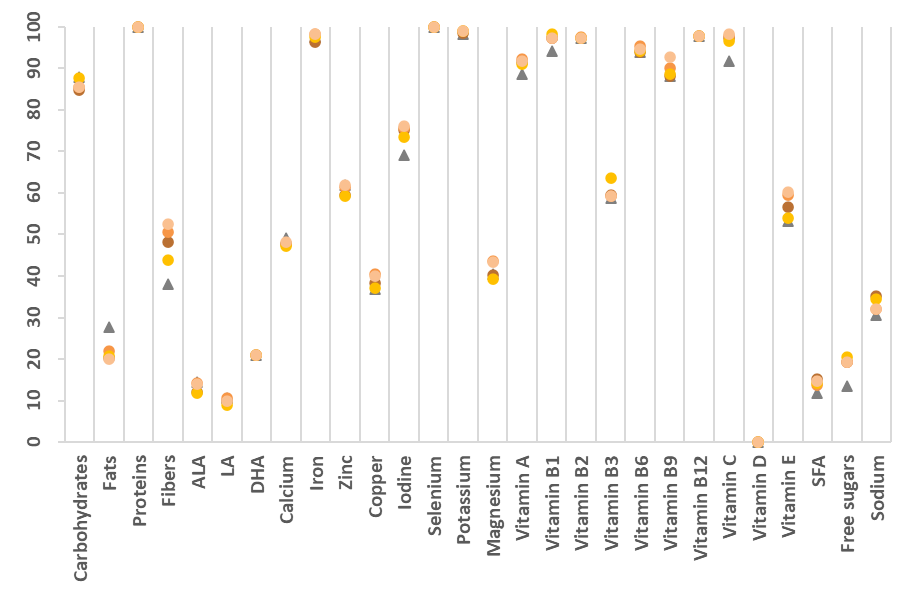 |
| 7-10 (n=481) | 11-17 (n=543) |
| 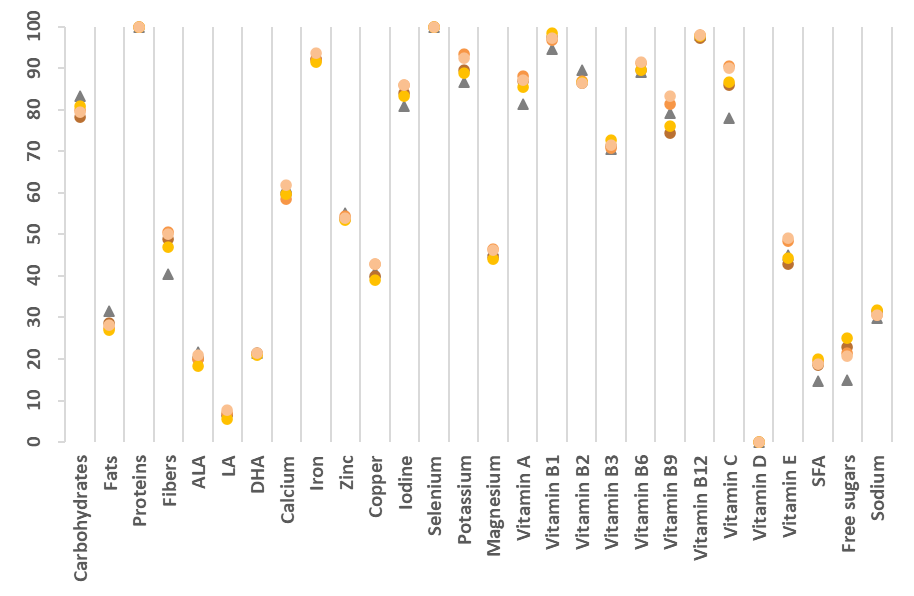 | 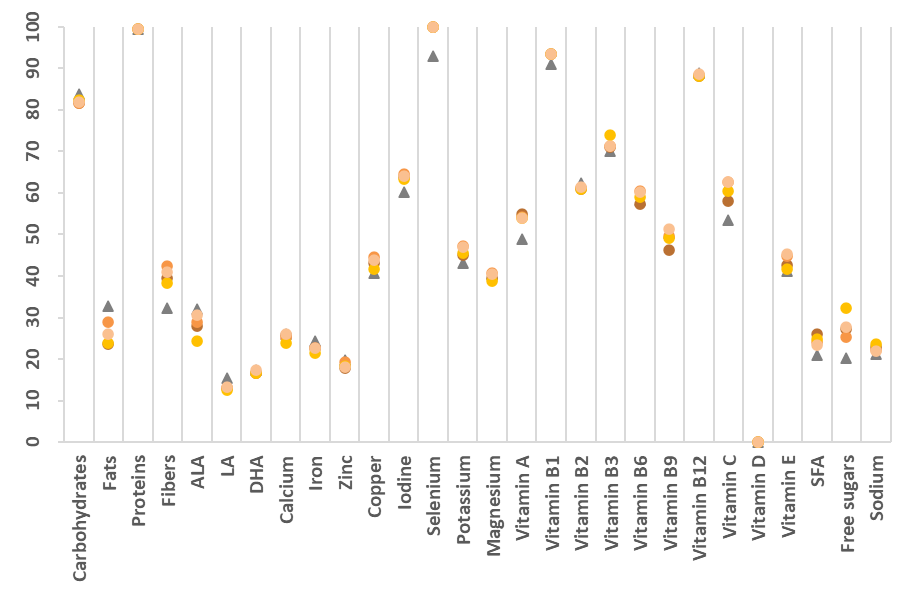 |
| 15-17 (n=406) |  |
| 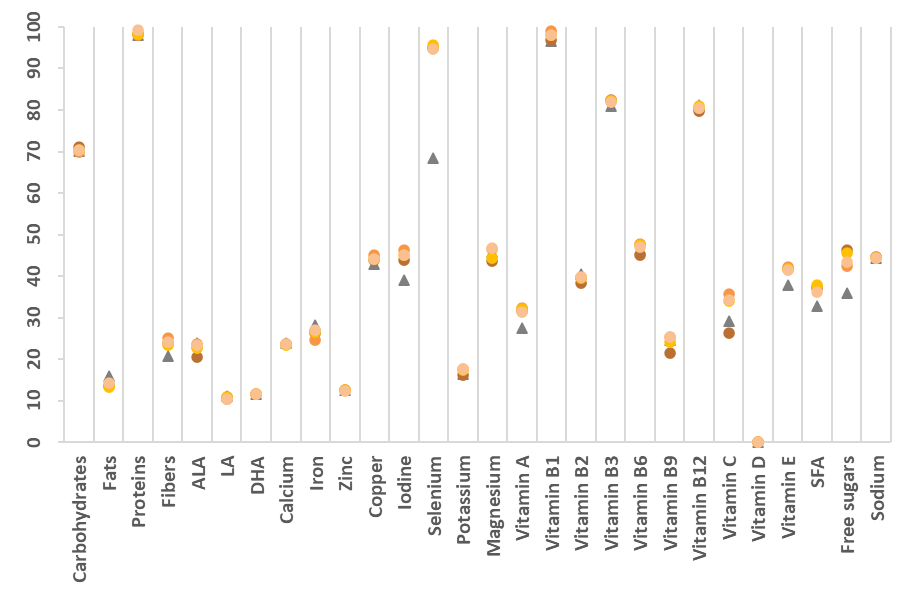 |  |
